# Morphological composition and fiber partitioning along regrowth in elephant grass CT115 intended for ethanol production

**DOI:** 10.1038/s41598-020-72169-2

**Published:** 2020-09-15

**Authors:** José A. Rueda, Juan de Dios Guerrero-Rodríguez, Sergio Ramírez-Ordoñes, Cecilio U. Aguilar-Martínez, Wilber Hernández-Montiel, Eusebio Ortega-Jiménez

**Affiliations:** 1 Universidad del Papaloapan, Campus Loma Bonita, Av. Ferrocarril SN Loma Bonita, CP 68400 Oaxaca, Mexico; 2 Colegio de Postgraduados Campus Puebla, Km 125.5 Boulevard Forjadores de Puebla, CP 72760 Puebla, Mexico; 3 Colegio de Postgraduados Campus Veracruz, Km 88.5 Carretera Xalapa-Veracruz, CP 91700 Veracruz, Mexico

**Keywords:** Field trials, Polysaccharides

## Abstract

Leaf share, plant age and growth season are often overlooked as modifiers of the biomass quality in energy crops. The current work studied the effect of the given factors on the biomass yield and the biomass quality in Elephant grass CT115, intended for bioethanol production, in Veracruz, Mexico. Two seasons per year, 5 months each, were tracked on a 2-weeks basis. The climate is warm wet with summer rains, 1,142 mm of annual rainfall, and 26 °C monthly temperature. From day 56 of the wet season or from day 84 of the dry season, stems accumulated 12 or 6 Mg ha^−1^, respectively, while green leaves increased only 1 Mg. Higher biomass quality was recorded for the leaf fraction, or for the wet season regrowth. For instance, lignin contained in stems meant twice that of leaves, whereas stems recorded 20% less lignin in the wet season as compared to the dry season. Despite holocellulose being similar between fractions or seasons, hemicellulose and cellulose showed inverse correlation, while lignin and cellulose contents were directly correlated in stems. Increasing the annual harvest of green leaves will improve biomass quality, which is known to increase biodegradability and might improve the annual ethanol yield.

## Introduction

Second-generation ethanol derives from lignocellulosic raw materials, while first-generation ethanol derives from starch rich crops like corn or from sucrose rich crops such as sugarcane^1^. Despite the industrial production of second-generation biofuels yet being incipient; substantial research is being conducted for both production and conversion of raw materials^2^. Among the energetic crops, perennial grasses stand as the leading alternative, as their use overcome the major drawbacks of both the fossil fuels and the first-generation biofuels. For instance, they can recapture in only months the greenhouse gases emitted to the atmosphere when the ethanol produced from them is burnt^3^, plus their utilization may prevent the use of crops essential to human nutrition^1^. Elephant grass, *Cenchrus purpureus* (Schumach.) Morrone, has been widely studied as energy crop for the production of lignocellulosic ethanol. This grass yields above 45 Mg ha^-1^ under low input systems, while it may endure many harvests a year, therefore meaning a continuous supply of biomass for the ethanol industry^4^.

In elephant grass, green leaves yield 10% more ethanol than stems^5^. Nonetheless, the share of green leaves decreases as the crop ages^6^, while the leaf accumulation pattern may differ between growth seasons^7,8^. Since leaves play a key role in ethanol yield, both the accumulation and the chemical constitution of green leaves are considered with special emphasis.

Leaf accumulation follows a definite pattern. For instance, (1) the number of leaves in elephant grass varies within a known range depending on its management^9^, such number corresponds to eight to eleven green leaves^6^. Accordingly, (2) the leaf yield should become stable when the appearance of new leaves synchronizes the death of the oldest^10^. Finally, (3) once leaf mass approaches the yield plateau, the grass resources will be allocated to stem elongation driven by intraspecific competition^9^, so that green leaf accumulation may grow slower, which might limit the ethanol yield. For instance, in elephant grass, an increase of 35 Mg ha^−1^ in overall yield occurs at unvarying green leaf mass; therefore, green leaf accumulation has a biologic limit far smaller than that of stem^6^.

Elephant grass is far from being a unique raw material whose biomass quality remains constant. Apart from the within variety broad variation, which is the reason we undertook the current research, elephant grass has an enormous genetic diversity. Some attributes that have served the purpose of identifying and discriminating among genotypes involve those related to the chemical composition^11^, as well as some morphological features such as plant height, and number of tillers^12^.

Analytical methods used in the field of ruminant nutrition have allowed to understand both the chemical composition and the biodegradability of energy crops intended for bioethanol production^13^. In such approach, the fiber content is measured as the fraction of feedstocks which is insoluble in neutral detergent (such fraction named NDF). The combined content of cellulose and lignin corresponds to the fraction recovered after diluting a sample in acid detergent (such fraction named ADF)^14^, and the lignin content (named ADL) is assessed as the remnant from dilution in sulfuric acid^15^. In addition, hemicellulose and cellulose contents are estimated by subtracting ADF from NDF, or ADL from ADF, respectively.

Bioethanol yield is directly correlated to in vitro* digestibility* of the dry matter and inversely correlated to ADF and ADL contents^5^. Furthermore, the content of lignin, inherent to stem aging, has been proposed as the main factor limiting fiber digestibility^16^. Accordingly, higher digestibility^17^ and lower lignin content^18^, both leading to higher ethanol yield, converge in the leaf fraction^5^. In elephant grass, the content of most fiber components increase as the plant ages^19^, whereas such content may differ within^19^ and between^7^ growth seasons.

Variations in biomass quality due to plant age, plant composition, and season of regrowth are often overlooked in research works dealing with conversion of grass crops for bioethanol production. For instance, they merely mention the grass species^20^ or the fraction^21^. In fact, most studies on morphological and chemical composition of elephant grass deal with few age classes, a target growth height, or a fixed cutting frequency. In order to fill that gap of knowledge, the present study closely tracks the accumulation pattern and the fiber partition in both leaves and stems of elephant grass CT115, throughout five months of undisturbed regrowth, during the wet and dry seasons.

## Results and discussion

### Morphological composition

**Figure 1:**
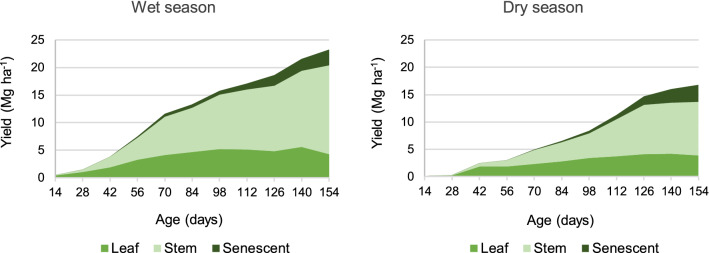
Yield by morphological fraction (piled to total yield) in elephant grass CT115, along 154 days of undisturbed regrowth, for the wet and dry seasons.

Yield is presented by season, fraction and regrowth age in Fig. 1. Overall biomass yield corresponds to the upper limit of the piled graphic. Leaf yield is shown at the base of the figure, in order to draw attention to the low relative variability in leaf yield across both seasons. When regrowth occurred under limiting weather conditions, leaf yield did not surpass 4 Mg ha^−1^. However, during the wet season, when higher soil moisture and higher temperature were available to promote regrowth, leaf yield reached 5 Mg ha^−1^. In growth cycles 154 days long, despite the leaf accumulation showing a biologic limit, stem accumulated 16 Mg ha^−1^ in the wet season or 10 Mg ha^−1^ in the dry season, whereas leaf proportion meant only 20% of the available biomass by day 154 in either season. Similar data for leaf proportion and leaf yield have been reported for elephant grass subjected to a single harvest per year^22^. However, management under long growth cycles implies reducing the annual harvest of green leaves across the year, as noticed in a previous work^23^.

Decisions on the utilization of elephant grass CT115 intended for ethanol production should focus on increasing the annual harvest of green leaves, in order to improve the yearly harvest of ethanol from a given field^17,23^. Cutting intervals under 70 days of regrowth might be established in order to prevent excessive stem accumulation. That in turn, according to a previous work, might increase both the leaf yield per harvest as well as the biomass yield per year^23^. The continuous stem accumulation, at relatively unvarying offer of green leaves, coincides with a previous study in which elephant grass is kept under undisturbed growth^6^. Furthermore, higher annual biomass yield has been reported for cutting intervals under three months, which also achieved a higher harvest of green leaves through the year^23^.

Cutting intervals around 70 days might prevent useless stem accumulation and reduce the fiber content of the harvested biomass, therefore promoting a higher biodegradability^24^. Longer cutting intervals have been associated to a higher stem growth, a higher plant lignification, and lower biodegradability^16^.

### Fiber partition as affected by season and fraction

**Figure 2:**
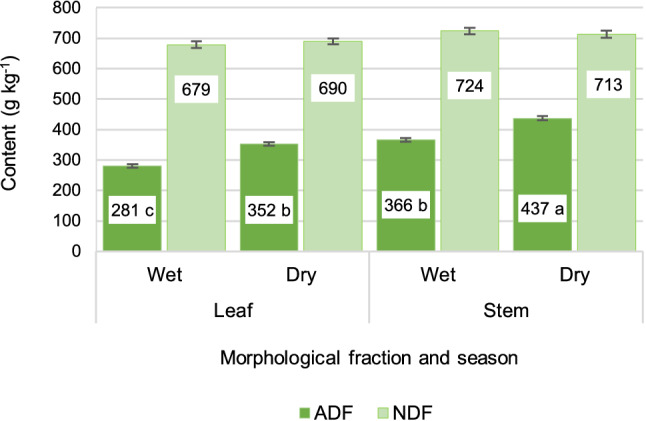
Acid detergent fiber (ADF) and neutral detergent fiber (NDF), averaged across 154 days of undisturbed regrowth, on a 14-days basis, in leaves and stems of elephant grass CT115, for the wet and dry seasons. Means with different letter are different at P < 0.0001 level, for ADF bars.

**Figure 3:**
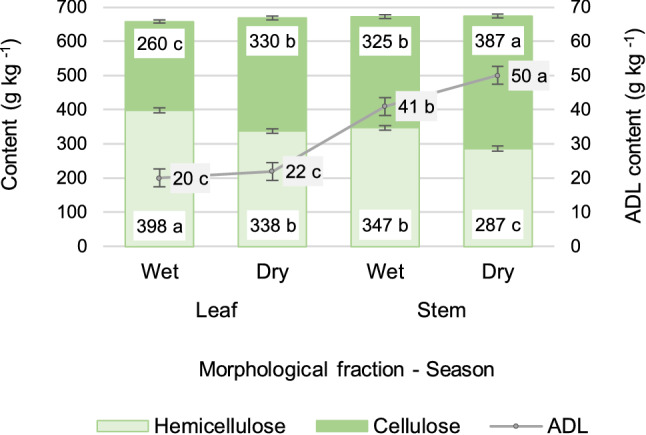
Hemicellulose, cellulose, holocellulose (combined-bar height), and acid detergent lignin (ADL) in leaves and stems of elephant grass CT115, during the wet and dry seasons. Average for eleven dates from day 14 to 154, on a 14-days basis. Means with different letter within component, are different at P < 0.0001 level, while for ADL differences are validated at P < 0.05 level. Standard error bars are indicated for every mean.

Neutral detergent fiber (NDF) and acid detergent fiber (ADF) contents are shown in Fig. 2, whereas cellulose, hemicellulose and acid detergent lignin (ADL) contents are shown in Fig. 3, both organized by morphological component and season. Across season, the season-fraction interaction was not significant for NDF (P = 0.99), ADF (P = 0.94), hemicellulose (P = 0.97), cellulose (P = 0.42), holocellulose (P = 0.71), ADL (P = 0.17) or ashes (P = 0.92) contents. In consequence, differences between seasons remain true within each fraction and differences between fractions remain true within each season. Leaf had 34 ± 10.8 g kg^−1^ less NDF (mean ± sed; P = 0.002), 85 ± 9.5 g kg^−1^ less ADF (P < 0.0001), 51 ± 7.1 g kg^−1^ more hemicellulose, 61 ± 5.1 g kg^−1^ less cellulose, 24 ± 2.7 g kg^−1^ less ADL, and 28 ± 5.9 g kg^−1^ more ashes, as compared to stem (P < 0.0001). In addition, the wet season regrowth showed similar NDF (P = 0.31) and ashes (P = 0.40) contents, but 71 ± 6.2 g kg^−1^ less ADF (P < 0.001), 60 ± 7.1 g kg^−1^ more hemicellulose, 66 ± 5.1 g kg^−1^ less cellulose (P < 0.0001) and 5.3 ± 2.7 g kg^−1^ less ADL (P < 0.05) than the dry season regrowth. For information about the adjustment and significance of each explicative variable on the model, refer to Supplementary Tables.

Green leaf meant higher hemicellulose, but lower cellulose and lignin contents than stems. In fact, a high biodigestibility of the dry matter has been reported for leaves, as compared to stems, for the grasses *Cynodon* sp., *Arundo donax,* and *Cenchrus purpureus*^5^. In addition, higher digestibility and higher protein content have been reported for the leaves of *Andropogon gayanus*^18^. A higher hemicellulose concurs with a lower lignification and higher content of non-fiber soluble components, which could be converted to ethanol. Furthermore, a great number of research works had been addressed to the conversion of hemicellulose to ethanol^25^.

The higher content of biodegradable compounds as well as the lower cellulose and lignin contents recorded for the wet season regrowth, coincides with a previous study where elephant grass was managed at a cutting interval of 8 weeks throughout the year^8^. In addition, a higher in vitro digestibility, which is related to higher ethanol production^5^, was reported for the grass *Andropogon gayanus* grown in the wet season, as compared to the dry season regrowth^18^, which might imply a lower cell wall content (NDF). On the other hand, a study about variations in the chemical constitution of elephant grass between seasons found higher quality for the dry season regrowth^7^. This finding, which diverges from the current study, may be due to the important differences in the rainfall distribution throughout the year, since Indonesia is located in the Equator, and rainfall occurs to some extent in every month.

Holocellulose content was similar for the leaf and stem fractions (663 ± 7.1 and 673 ± 7.5, P = 0.35). Likewise, it was similar for the wet and dry seasons (665 ± 7.3, 671 ± 7.3, P = 0.58). The given similarities occurred despite the wide inverse variations in cellulose and hemicellulose contents both between fractions and between seasons (Fig. 3). The leaf from the wet season averaged 138 g kg^−1^ more hemicellulose than cellulose, and the stem from the dry season showed 100 g kg^−1^ more cellulose than hemicellulose. Surprisingly, hemicellulose and cellulose contents were similar between the stem from the wet season and the leaf from the dry season (Fig. 3).

### Fiber partition as affected by plant age

Fluctuations in NDF, ADF, ADL and ashes contents across each season are shown in Table 1, by morphological fraction: leaf and stem. In both seasons leaf fraction recorded less ADF since day 42 (except contents were alike on day 56 of the wet season), less ADL since day 70, and more ashes since day 56 or 42 of the wet and dry seasons, respectively. During the first 98 days of regrowth, NDF and ADF contents followed increasing trends in either season or either fraction; while they remained constant afterwards. ADL content increased through day 98 for the stem fraction in either season, whereas it remained constant for the leaf fraction, in both seasons, with one exception. (Table 1). Ash content declined across the two seasons, but decreased slower in leaf fraction.

**Table 1:** Neutral detergent fiber (NDF), acid detergent fiber (ADF), acid detergent lignin (ADL) and ashes contents in leaves and stems of elephant grass CT115, across 154 days of undisturbed regrowth, for the wet and dry seasons.

	NDF (g kg^−1^)		ADF (g kg^−1^)		ADL (g kg^−1^)		Ashes (g kg^−1^)	
	Leaf	Stem	Leaf	Stem	Leaf	Stem	Leaf	Stem
**Wet season**								
14	539^d^	–	227^b^	–	23	–	123^a^	–
28	619^Ac^	593^Ad^	269^Aab^	283^Ac^	25^A^	24^Ae^	78^Bb^	130^Aa^
42	693^Aab^	690^Ac^	305^Ba^	333^Ab^	20^A^	15^Ae^	60^Bc^	69^Ab^
56	680^Bb^	729^Aabc^	309^Ba^	366^Aab^	31^A^	38^Abcd^	59^Ac^	44^Bc^
70	700^Aab^	711^Abc^	269^Bab^	380^Aab^	16^B^	34^Acd^	63^Abc^	24^Bd^
84	705^Aab^	715^Abc^	273^Bab^	393^Aa^	17^B^	43^Aabc^	65^Abc^	22^Bd^
98	691^Aab^	702^Ac^	284^Ba^	410^Aa^	18^B^	57^Aa^	72^Ab^	26^Bd^
112	720^Aab^	735^Aabc^	302^Ba^	375^Aab^	18^B^	54^Aa^	66^Abc^	33^Bcd^
126	733^Ba^	771^Aa^	300^Ba^	384^Aa^	19^B^	44^Aabc^	65^Abc^	32^Bcd^
140	700^Bab^	755^Aab^	289^Ba^	374^Aab^	20^B^	50^Aabc^	65^Abc^	24^Bd^
154	692^Bab^	733^Aabc^	262^Bab^	366^Aab^	19^B^	53^Aab^	67^Ab^	20^Bd^
se	8.6	8.6	8.2	8.2	3.2	3.2	2.8	2.8
R^2^	0.98	0.98	0.98	0.98	0.96	0.96	0.99	0.99
**Dry season**								
14	622^b^	–	328^b^	–	28^ab^	–	118^a^	–
28	627^Bb^	662^Ab^	339^Bab^	404^Acd^	26^Aab^	27^Ad^	112^Ba^	130^Aa^
42	630^Bb^	723^Aab^	341^Bab^	419^Abcd^	25^Aab^	21^Ad^	85^Ab^	70^Bb^
56	703^Aab^	666^Ab^	382^Aab^	389^Ad^	26^Aab^	29^Acd^	77^Abcd^	63^Bb^
70	748^Aa^	751^Aab^	393^Ba^	456^Aabc^	32^Ba^	44^Ac^	70^Acde^	49^Bc^
84	681^Aab^	689^Ab^	350^Bab^	417^Abcd^	27^Bab^	49^Ab^	79^Abc^	47^Bc^
98	737^Ba^	798^Aa^	368^Bab^	487^Aa^	20^Bab^	63^Aab^	68^Adef^	30^Bd^
112	729^Aab^	733^Aab^	347^Bab^	442^Aabc^	12^Bb^	60^Aab^	60^Aefg^	32^Bd^
126	706^Bab^	760^Aab^	341^Bab^	464^Aab^	17^Bab^	66^Aa^	57^Afg^	21^Bde^
140	713^Aab^	705^Aab^	352^Bab^	432^Abcd^	17^Bab^	69^Aa^	52^Ag^	19^Be^
154	698^Bab^	756^Aab^	339^Bab^	462^Aab^	13^Bb^	74^Aa^	53^Ag^	20^Be^
se	18.0	18.0	9.8	9.8	2.9	2.9	1.9	1.9
R^2^	0.87	0.87	0.96	0.96	0.98	0.98	0.99	0.99

*se* standard error, *R*^*2*^ model adjustment.

^a,b,…g^Means in the same column with different lowercase letter are different (P < 0.05).

^A,B^Means in the same row and variable with different uppercase letter are different (P < 0.05).

**Figure 4:**
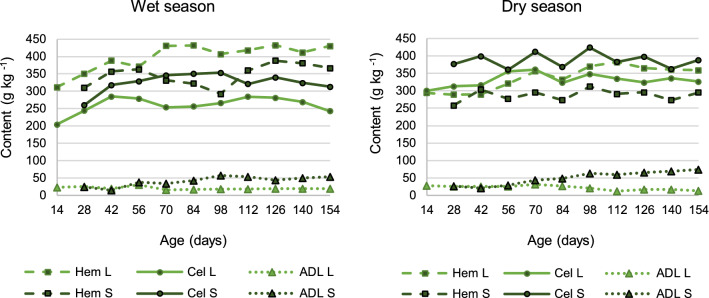
Within season variation in hemicellulose (Hem), cellulose (Cel), and acid detergent lignin (ADL) contents in leaves (L) and stems (S) of elephant grass CT115, during 154 days of undisturbed regrowth for the wet and dry seasons (starting in June or December, respectively).

Variations in hemicellulose, cellulose and ADL contents within each season are presented in Fig. 4, ordered by season, morphological fraction and age. Actual means and statistical differences for the visual information of such figure, are presented in Table 2. The higher hemicellulose and lower cellulose contents recorded for the leaf fraction across each season (Fig. 3) remained true virtually on every age in either season.

**Table 2:** Hemicellulose, cellulose and holocellulose contents in leaf and stem of elephant grass CT115, across 154 days of undisturbed regrowth, for the wet and dry seasons.

	Hemicellulose (g kg^−1^)		Cellulose (g kg^−1^)		Holocellulose (g kg^−1^)	
	Leaf	Stem	Leaf	Stem	Leaf	Stem
**Wet season**						
14	312^e^	–	204^c^	–	516^e^	–
28	350^Ad^	310^Bde^	244^Ab^	260^Ac^	594^Ad^	570^Ad^
42	388^Abc^	357^Babc^	285^Ba^	318^Aab^	673^Abc^	675^Abc^
56	371^Acd^	363^Bab^	279^Bab^	329^Aab^	649^Bc^	692^Ab^
70	431^Aa^	331^Bbcd^	254^Bab^	346^Aab^	684^Aab^	677^Abc^
84	432^Aa^	322^Bcde^	256^Bab^	350^Aab^	688^Aab^	672^Abc^
98	407^Aab^	292^Be^	266^Bab^	353^Aa^	673^Abc^	645^Ac^
112	418^Aab^	360^Bab^	284^Ba^	321^Aab^	702^Aab^	681^Ab^
126	433^Aa^	388^Ba^	281^Bab^	340^Aab^	714^Aa^	727^Aa^
140	412^Aab^	381^Ba^	269^Bab^	324^Aab^	680^Aabc^	705^Aa^
154	430^Aa^	367^Bab^	243^Bbc^	313^Ab^	673^Abc^	680^Abc^
se	6.4	6.4	6.7	6.7	7.2	7.2
R^2^	0.98	0.98	0.97	0.97	0.98	0.98
**Dry season**						
14	294^b^	–	300^c^	–	594^b^	–
28	289^Ab^	258^B^	313^Bbc^	377^Abc^	602^Ab^	635^Ab^
42	290^Ab^	304^A^	316^Babc^	399^Aabc^	606^Bab^	703^Aab^
56	321^Aab^	277^B^	356^Aab^	361^Ac^	677^Aa^	637^Ab^
70	356^Aab^	295^B^	361^Ba^	412^Aab^	716^Aa^	707^Aab^
84	332^Abc^	273^B^	323^Babc^	368^Abc^	654^Aab^	640^Ab^
98	370^Aa^	312^B^	348^Bab^	424^Aa^	717^Aa^	735^Aa^
112	382^Aa^	291^B^	335^Babc^	383^Aabc^	717^Aa^	674^Aab^
126	365^Aa^	296^B^	324^Babc^	398^Aabc^	689^Aa^	694^Aab^
140	361^Aab^	273^B^	336^Babc^	363^Ac^	696^Aa^	636^Ab^
154	359^Aab^	295^B^	326^Babc^	388^Aabc^	685^Aa^	682^Aab^
se	10.7	10.7	8.0	8.0	16.4	16.4
R^2^	0.92	0.92	0.95	0.95	0.86	0.86

*se* standard error, *R*^*2*^ model adjustment.

^a,b,c…g^Means in the same column with different lowercase letter are different (P < 0.05).

^A,B^Means in the same row and variable, with different uppercase letter are different (P < 0.05).

Hemicellulose content = NDF – ADF, cellulose content = ADF – ADL, and holocellulose = hemicellulose + cellulose.

For the leaf fraction, hemicellulose content increased through day 70 of the wet season or through day 98 of the dry season, whereas the cellulose content increased through day 42 for the leaf fraction, in either season, and kept on similar records from then onwards. Regarding stem fraction, hemicellulose content increased only during the wet season, through day 56, then decreased, but it reached a second maximum on day 126. Cellulose content was relatively constant in either season for the stem fraction, but reached a maximum on day 98 in both seasons.

The similar holocellulose content found between leaf and stem fractions on average across seasons (Fig. 3), remained true in ten out of the eleven ages, in either season. This was especially interesting, given that hemicellulose and cellulose contents differed between leaf and stem, virtually on every age class (Table 2).

A higher hemicellulose content for the leaf of elephant grass has been previously reported for the dry season, while a higher cellulose content has only been reported for the wet season; both results in a study in Thailand^26^, as an average for eight varieties of elephant grass. Climate and variety differences explain the discrepancies with the current work.

Published data are consistent with the fact that grass age and the content of most of the fiber constituents are directly related^19,27,28^; nonetheless, just a few age classes are usually included. Hemicellulose content has been reported to decrease for the whole plant of elephant grass in long-lasting growth cycles^19^. The current study gives rationale for such fact, since along regrowth, an increment of the stem proportion (Fig. 1), whose hemicellulose content was lower (Table 2), will lead to a lower hemicellulose content for the whole plant (see Supplementary Tables).

All seven variables describing chemical constitution in the current research work showed similar records from day 98 onwards, in each season and for each morphologic fraction.

### Correlation between fiber fractions

Hemicellulose and cellulose contents were inversely correlated across the whole data (r = − 0.58, P < 0.001), while such inverse correlation remained true within the leaf (r = − 0.34, P < 0.026) or stem (r = − 0.48, P = 0.001), as well as for the wet season alone (r = − 0.33, P = 0.033); while a similar trend occurred for the dry season (r = − 0.28, P < 0.071).

ADL and cellulose contents were directly correlated for the whole data (r = 0.57, P < 0.001) and for the stem fraction (r = 0.387, P = 0.015), but not for the leaf fraction (r = 0.12, P < 0.42). Finally, ADL was inversely correlated with hemicellulose content for the whole data (r = − 0.47, P < 0.001), or for the leaf fraction (r = − 0.54, P < 0.001), but not for to stem fraction (r = − 0.09, P < 0.54).

### Recommendations

Biomass quality of elephant grass CT115 can be improved, by means of increasing both the share of green leaves and the share of the wet season regrowth, in the biomass harvested along the year. A higher biomass quality, will in turn increase the annual yield of ethanol per area unit.

Strategies to accomplish a higher quality of the harvested biomass, as proposed above, may involve (1) cutting intervals of around 56 days during the wet season or around day 70 during the dry season, and (2) reduce cutting intensity. The latter implies cutting to a greater height, so that the fodder left uncut in the field will facilitate a faster restoration of the grass photosynthetic structures^27^. Nonetheless, such strategies may require further validation according to the wide diversity in climate conditions and crop management systems.

## Conclusions

Elephant grass CT115 must be harvested by day 56 of the wet season or by day 70 of the dry season, in order to increase both the share of green leaves per harvest and the annual yield of green leaves.

Green leaves recorded more hemicellulose, less cellulose and less lignin than stems, whereas the regrowth from the wet season recorded more hemicellulose, less cellulose and less lignin, than that from the dry season. Holocellulose content was similar between leaves and stems of elephant grass, as well as between the regrowth from the wet season and that from the dry season.

When elephant grass is cultivated as energy crop for conversion to ethanol, leaves should be preferred over stems, whereas the wet season regrowth should be preferred over that from the dry season.

Age is the main factor affecting the chemical composition of elephant grass. Cutting intervals around 56 days for the wet season or around 70 days for the dry season provide an acceptable yield—quality balance. Longer intervals would sacrifice biomass quality, while shorter ones would sacrifice yield. The lower biomass quality of the late regrowth is explained in terms of both higher stem share and higher stem lignification.

Hemicellulose and cellulose contents were inversely correlated. In addition, ADL and hemicellulose contents were inversely correlated for the leaf fraction, while ADL and cellulose contents were directly correlated for the stem fraction.

## Methods

### Location and weather

The field assay was conducted at the Papaloapan Site of the Mexican Institute for Forestry, Agricultural and Livestock Research (INIFAP), Veracruz, Mexico. Climate is warm-wet with a summer rainy season, 1,142 mm of annual rainfall, and 25.8 °C of monthly temperature^29^. Chemical determinations were carried out at the Laboratory of the Papaloapan University. Data for the present manuscript are permanently available online^30^.

### Planting and experimental conditions

This study was undertaken after establishment assessment^31^ and simultaneously to a tiller population dynamics study^32^, so that field methods for establishment are widely explained in such studies. Data collection for the wet seasons started on June 15, 2013 and June 7, 2014, while the dry season cycle started on December 7, 2013. The experimental unit was a plot with six rows, 8 m long and 0.6 m apart, for an area of 28.8 m^2^ by plot, and an effective plot of 16.8 m^2^, once discarding edges. Plots were distributed in four fully randomized blocks, given some field heterogeneity, two replications within every block were used. According to the Cotaxtla Laboratory of INIFAP, the soil is an orthic acrisol with sandy loam texture^33^, characterized by low organic matter content (0.34%), acidic pH (3.5), and limited contents of N, Ca, Mg and Cu.A 200:100:200 N, P and K (kg ha^−1^ year^−1^) fertilization formula was applied manually, half in the second week of regrowth, and the remaining half in the eighth week.

### Yield and morphological composition

Yield and morphological composition were estimated as explained in a previous work^31^, but methods are summarized here. From day 14 through day 154, and on a 2-week basis, a sample was cut from a 2 m long section of a central row, at 20 cm high, then weighed and recorded as *sample fresh weight*. A subsample was drawn from the sample and split into morphological fractions: stem, leaf and dead material. The fractions were placed on a paper bag tagged age, plot and fraction, and its weight was recorded *the fraction fresh weight*. The stems were sliced to 2 cm to enable drying. Paper bags were placed in a forced air oven for 96 h at 65 °C and their weights were recorded as *the fraction dry weight*. Leaf, stem and dead material weights, combined within the plot, were added to obtain *the subsample weight*, first in fresh and then in dry basis. The *fresh* and *dry* weights were used to estimate the dry matter yield for each morphological fraction, while *the subsample fresh* and *dry weight*s were extrapolated, first to *the sample* and then to a hectare, in order to estimate the dry matter yield (Mg ha^−1^).

### Chemical composition

Oven-dried samples of stem and leaf were ground to 1 mm in a Wiley mill. A 2 g sample was fully dried at 105 °C for 4 h to estimate dry matter, so that the contents could be expressed on dry basis. Ash content was measured by oven incineration of 1 g sample at 600 °C for 2 h^34^.

Neutral detergent fiber (NDF) and acid detergent fiber (ADF) contents were sequentially assessed using an Ankom 200 fiber analyzer^14^. Acid detergent lignin (ADL) content was measured by dilution in sulfuric acid^15^. Cellulose was estimated by subtracting ADF from NDF, hemicellulose was estimated by subtracting ADL from ADF, and holocellulose was obtained by adding cellulose and hemicellulose.

### Statistical analyses

Two models of analyses of variance were run for the fiber components. The first model included the whole data at once (Eq. 1), while the second model was used to study the variance occurring within each of the two seasons, separately (Eq. 2).

1 $$Y_{ijk} = \mu + S_{i} + F_{j} + SF_{ij} + \varepsilon_{ijk}$$

2 $$Y_{ijk} = \mu + A_{i} + F_{j} + AF_{ij} + \varepsilon_{ijk}$$

Accordingly: $Y_{ijk}$: data point or measurement, $\mu$: overall mean for each given variable, $S_{i}$: growth season, $F_{j}$: morphological fraction, $SF_{ij}$: season-by-fraction interaction, and $\varepsilon_{ijk}$: error term for each data point. In the second model (Eq. 2), the given definitions remain; plus, the effects $A_{i}$: age, and $AF_{ij}$: age-by-fraction interaction. Analyses of variance were run by the MIXED procedure, and Tukey tests were carried out for the comparison between means. Finally, Pearson correlation coefficients were estimated between the variables regarding chemical composition. All three tests were run in SAS 9.4^35^.

## Supplementary information

Supplementary Tables.

## Data Availability

Data for the present manuscript has been made public and properly cited in the manuscript in Reference 16 (Rueda, J. A. Cenchrus purpureus. Figshare Dataset 2019). https://doi.org/10.6084/m9.figshare.11354201.v2.
