# Supplementary material for: Morphological composition and fiber partitioning along regrowth in elephant grass CT115 intended for ethanol production
Source: Sci Rep. 2020 Sep 15;10:15118. doi: 10.1038/s41598-020-72169-2 (PMC7492190; doi:10.1038/s41598-020-72169-2)
Supplement: Supplementary file 1 — Supplementary Tables. [file 41598_2020_72169_MOESM1_ESM.docx]

_________________________________________________________________________

**"Morphological composition and fiber partitioning along regrowth in elephant grass CT115 intended for ethanol production"**

**by**: José A. Rueda*, Juan de Dios Guerrero-Rodríguez*, Sergio Ramírez-Ordoñes, Cecilio U. Aguilar-Martínez, Wilber Hernández-Montiel & Eusebio Ortega-Jiménez*

The current Supplementary Material includes 4 tables, with the purpose of including all means plus their statistical comparisons, which cannot be given in the figures.

**Table 1.**  Model adjustment (R^2^), partial R^2^ for each explicative variable, and observed probabilities of type I error, for the variables of fiber composition of elephant grass CT115, according to the model: Content (in g kg^-1^) = Fraction + Season + Interaction.

|  |  | Fraction | | Season | | Interaction | |
| --- | --- | --- | --- | --- | --- | --- | --- |
|  | R^2^ | P | R^2^ | P | R^2^ | P | R^2^ |
| NDF | 0.12 | P=0.002 | 0.11 | P=0.3 | 0.01 | P=0.99 | 0.00 |
| ADF | 0.80 | P<0.001 | 0.47 | P<0.001 | 0.33 | P=0.94 | 0.00 |
| ADL | 0.52 | P<0.001 | 0.49 | P<0.001 | 0.02 | P=0.17 | 0.01 |
| Ashes | 0.22 | P<0.001 | 0.22 | P=0.41 | 0.00 | P=0.92 | 0.00 |
| Hemicellulose | 0.60 | P<0.001 | 0.25 | P<0.001 | 0.35 | P=0.97 | 0.00 |
| Cellulose | 0.78 | P<0.001 | 0.36 | P<0.001 | 0.42 | P=0.42 | 0.00 |
| Holocellulose | 0.01 | P=0.35 | 0.01 | P=0.58 | 0.00 | P=0.71 | 0.00 |

NDF: Neutral detergent fiber, ADF: Acid detergent fiber, ADL: Acid detergent lignin, H: Hemicellulose, C: Cellulose, HC: Holocellulose. The model had two levels for fraction: leaf and stem, and two levels for season: wet and dry.

**Table 2.** Fiber partition in leaves or stems of elephant grass CT115, as an average of 5 months of undisturbed regrowth, in a two season a year management (g kg^-1^).

|  |  | Leaves | Stems | *Season mean* |
| --- | --- | --- | --- | --- |
|  | Season |  |  |  |
| NDF |  |  |  |  |
|  | Wet | 679 ± 10.5 ^b^ | 713 ± 11.0 ^ab^ | 696 ± 7.6 |
|  | Dry | 690 ± 10.5 ^ab^ | 724 ± 11.0 ^a^ | 707 ± 7.6 |
|  | *Fraction mean* | 684 ± 7.4 ^B^ | 719 ± 7.8 ^A^ |  |
| ADF |  |  |  |  |
|  | Wet | 281 ± 6.1 ^c^ | 366 ± 6.4 ^b^ | 323 ± 4.4 ^B^ |
|  | Dry | 352 ± 6.1 ^b^ | 437 ± 6.4 ^a^ | 394 ± 4.4 ^A^ |
|  | *Fraction mean* | 316 ± 4.3 ^B^ | 402 ± 4.5 ^A^ |  |
| ADL |  |  |  |  |
|  | Wet | 20 ± 2.6 ^b^ | 41 ± 2.7 ^a^ | 31 ± 1.9 ^B^ |
|  | Dry | 22 ± 2.6 ^b^ | 50 ± 2.7 ^a^ | 36 ± 1.9 ^A^ |
|  | *Fraction mean* | 21 ± 1.9 ^B^ | 46 ± 1.9 ^A^ |  |
| Ashes |  |  |  |  |
|  | Wet | 71±5.7 | 42±6.0 | 56.6±4.2 |
|  | Dry | 75±4.2 | 48±6.0 | 61.5±4.2 |
|  | *Fraction mean* | 73±4.0 ^A^ | 45±4.2 ^B^ |  |
| Hemicellulose |  |  |  |  |
|  | Wet | 398 ± 6.9 ^a^ | 347 ± 7.3 ^b^ | 373 ± 5.0 ^A^ |
|  | Dry | 338 ± 6.9 ^b^ | 287 ± 7.3 ^c^ | 312 ± 5.0 ^B^ |
|  | *Fraction mean* | 368 ± 4.9 ^A^ | 317 ± 5.1 ^B^ |  |
| Cellulose |  |  |  |  |
|  | Wet | 260 ± 5.0 ^c^ | 325 ± 5.3 ^b^ | 293 ± 3.6 ^B^ |
|  | Dry | 330 ± 5.0 ^b^ | 387 ± 5.3 ^a^ | 359 ± 3.6 ^A^ |
|  | *Fraction mean* | 295 ± 3.5 ^B^ | 356 ± 3.7 ^A^ |  |
| Holocellulose | |  |  |  |
|  | Wet | 658 ± 10.1 | 672 ± 10.6 | 665 ± 7.3 |
|  | Dry | 668 ± 10.1 | 674 ± 10.6 | 671 ± 7.3 |
|  | *Fraction mean* | 663 ± 7.1 | 673 ± 7.5 |  |

^A,B^ : Whether for fractions or seasons, means with different uppercase letter show statistical difference (Tukey, P<0.0001; except for NDF whose P=0.002 ).

^a,b,^ : Means within the interaction fraction-season with different lowercase letter, show statistical difference (Tukey, P<0.05). Hemicellulose = NDF – ADF, Cellulose= ADF – ADL. Holocellulose = cellulose + hemicellulose. Each mean followed by its standard error.

**Table 3.** Model adjustment (R^2^), partial R^2^ for each explicative variable, and observed probabilities of type I error, for the variables associated to fiber composition in elephant grass CT115, according to the model: Content (in g kg^-1^) = Fraction + Age + Interaction.

| Season | Fiber fraction | R^2^ | Fraction | | Age | | Interaction | |
| --- | --- | --- | --- | --- | --- | --- | --- | --- |
|  |  |  | P | R^2^ | P | R^2^ | P | R^2^ |
| Wet | NDF | 0.90 | P<0.001 | 0.03 | P<0.001 | 0.82 | P=0.003 | 0.05 |
| season | ADF | 0.86 | P<0.001 | 0.56 | P<0.001 | 0.19 | P<0.001 | 0.11 |
|  | ADL | 0.94 | P<0.001 | 0.50 | P<0.001 | 0.18 | P<0.001 | 0.26 |
|  | Ashes | 0.91 | P<0.001 | 0.15 | P<0.001 | 0.51 | P<0.001 | 0.25 |
|  | Hemicellulose | 0.97 | P<0.001 | 0.35 | P<0.001 | 0.47 | P<0.001 | 0.15 |
|  | Cellulose | 0.85 | P<0.001 | 0.50 | P<0.001 | 0.26 | P<0.001 | 0.09 |
|  | Holocellulose | 0.96 | P=0.842 | 0.00 | P<0.001 | 0.91 | P=0.001 | 0.05 |
|  |  |  |  |  |  |  |  |  |
| Dry | NDF | 0.82 | P<0.001 | 0.07 | P<0.001 | 0.61 | P=0.047 | 0.14 |
| season | ADF | 0.89 | P<0.001 | 0.65 | P<0.001 | 0.13 | P<0.001 | 0.11 |
|  | ADL | 0.98 | P<0.001 | 0.51 | P<0.001 | 0.13 | P<0.001 | 0.34 |
|  | Ashes | 0.93 | P<0.001 | 0.14 | P<0.001 | 0.72 | P<0.001 | 0.07 |
|  | Hemicellulose | 0.91 | P<0.001 | 0.44 | P<0.001 | 0.34 | P=0.004 | 0.13 |
|  | Cellulose | 0.85 | P<0.001 | 0.56 | P<0.001 | 0.19 | P=0.002 | 0.10 |
|  | Holocellulose | 0.86 | P=0.840 | 0.00 | P<0.001 | 0.63 | P=0.006 | 0.23 |

NDF: Neutral detergent fiber, ADF: Acid detergent fiber and ADL: Acid detergent lignin. The model has two levels for fraction: leaf and stem, and eleven levels for age: from day 14 through day 154 on a 14-days basis.

**Table 4**. Pearson correlation coefficients between the variables of fiber composition and ash content in elephant grass CT115, plus type I error.

|  | ADF | ADL | Hemicellulose | Cellulose | Holocellulose | Ashes |
| --- | --- | --- | --- | --- | --- | --- |
|  |  |  |  |  |  |  |
| NDF | 0.62  <.0001 | 0.44  <.0001 | 0.26  .0017 | 0.61  <.0001 | 0.94  <.0001 | **-**0.74  <.0001 |
| ADF |  | 0.75  <.0001 | **-**0.60  <.0001 | 0.97  <.0001 | 0.400  .0002 | **-**0.54  <.0001 |
| ADL |  |  | **-**0.47  <.0001 | 0.58  <.0001 | 0.11  0.325 | -0.62  <.0001 |
| Hemicellulose |  |  |  | **-**0.58  <.0001 | 0.46  <.0001 | **-**0.09  .432 |
| Cellulose |  |  |  |  | 0.46  <.0001 | **-**0.45  <.0001 |
| Holocellulose |  |  |  |  |  | **-**0.58  <.0001 |

NDF: Neutral detergent fiber, ADF: Acid detergent fiber and ADL: Acid detergent lignin.
